# Novel Insights into the Molecular Mechanisms of Chicken Breast Muscle Development by Integrating Non-Coding RNA and mRNA Profiles

**DOI:** 10.3390/ijms26178181

**Published:** 2025-08-23

**Authors:** Yuting Jin, Jie Dong, Jiahua Li, Minjie Huang, Deqian Wang, Xiaodong Tan

**Affiliations:** 1 Institute of Animal Husbandry and Veterinary Science, Zhejiang Academy of Agricultural Sciences, Hangzhou 310021, China; jinyvting1@163.com (Y.J.); dongj@zaas.ac.cn (J.D.);; 2 Key Laboratory of Livestock and Poultry Resources (Poultry) Evaluation and Utilization, Ministry of Agriculture and Rural Affairs, Hangzhou 310021, China

**Keywords:** chicken, lncRNA, circRNA, muscle development, weighted gene co-expression network analysis

## Abstract

Chicken meat represents the most widely consumed source of animal protein globally. The identification of non-coding RNAs (ncRNAs) that affect muscle development provides new selection targets for poultry breeding. In this study, muscle samples from high- and low-breast-weight chickens were collected and sequenced for long non-coding RNAs (lncRNAs), circular RNAs (circRNAs), and mRNAs. Using weighted gene co-expression network analysis, we found 95 lncRNAs and 46 circRNAs that were significantly associated with breast muscle traits. Subsequently, 51 candidate lncRNAs and 22 candidate circRNAs were screened through differential expression analysis. Finally, by constructing an ncRNA–mRNA regulatory network and performing pathway enrichment analysis, we identified four lncRNAs (e.g., MSTRG.9172.1) and seven circRNAs (e.g., novel_circ_009419) as key regulatory molecules. Functional analysis revealed that these molecules modulate genes such as *CD28*, *CCND2*, *TIAM1*, and *RRM2* through pathways including the actin cytoskeleton, p53 signaling pathway, and other pathways. In conclusion, this study provides clearer insight into the epigenetic regulatory network involved in chicken breast muscle development and offers important molecular markers for chicken genetic selection.

## 1. Introduction

Skeletal muscle development is a tightly regulated process involving intricate transcriptional networks, with poultry species serving as both agriculturally vital livestock and valuable models for myogenesis research [1]. During the embryonic development stage, myoblasts form primary and secondary muscle fibers through proliferation, differentiation, migration, and fusion, and the total number of muscle fibers is essentially fixed by 20 embryonic days of age [2]. After birth, muscle development in poultry is dominated by myofiber hypertrophy [3]. Throughout muscle growth and development, multiple genes play critical roles in muscle precursor cell proliferation, myofiber formation, and muscle function, including myogenic regulatory factor 5 (*Myf5*), myogenic determination factor (*MyoD*), myogenic regulatory factor 4 (*MRF4*), and myogenin (*MYOG*) [4]. These GRC genes have been widely demonstrated to directly regulate muscle development, but their upstream regulation involves an even more complex epigenetic network that fine-tunes gene expression to precisely control muscle development. Currently, research on these epigenetic regulators (e.g., long non-coding RNAs, circular RNAs) remains relatively limited, warranting further in-depth exploration.

Long non-coding RNAs (lncRNAs) are a class of non-coding RNAs longer than 200 nucleotides and are categorized as intergenic long-chain non-coding RNAs, antisense long-chain non-coding RNAs, and bidirectional long-chain non-coding RNAs, among other categories [5]. They are widely involved in regulating muscle growth and development by encoding micropeptides, regulating transcription, and through other mechanisms [6]. Numerous studies have demonstrated that a variety of lncRNAs play important regulatory roles in chicken skeletal muscle development [7,8]. For example, Cai et al. reported that lncRNA (MYH1G-AS) could promote myoblast proliferation but inhibit myoblast differentiation [9]. In addition, some lncRNAs act as transcriptional regulators that can regulate protein-coding gene expression either by cis-acting or trans-acting mechanisms [10]. lncMAAT can either increase the expression of the neighboring gene Mbnl1 through cis-action or regulate the binding ability of the transcription factor SOX6 to miR-29b through trans-action, thus affecting the muscle atrophy process [11]. Despite the identification of numerous muscle-related genes, research on their upstream epigenetic regulation remains limited, with merely a fraction of lncRNAs uncovered.

Circular RNAs (circRNAs), a class of non-coding RNAs lacking 5′ caps and 3′ tails, possess a covalently closed-loop structure [12,13]. CircRNAs have a wide range of tissue- and cell-specific expression characteristics and play key roles in several physiological and pathological processes, including skeletal muscle development, neuronal function maintenance, ovarian development regulation, and osteoarthritis pathological processes [14,15,16,17]. In poultry, circRNAs can regulate chicken skeletal muscle development through multiple mechanisms, including a competitive endogenous RNA mechanism as microRNA (miRNA) sponges [18]. circFNDC3AL, which upregulates *BCL* expression by binding to miR-204, promotes the proliferation and differentiation of chicken skeletal muscle satellite cells [19,20]. Chen et al. reported that circHIPK3 promotes chicken embryonic skeletal muscle development and promotes the proliferation and differentiation of myoblasts [21]. In addition, circRNAs can also participate in regulating muscle development by modulating source genes. Similarly to findings on lncRNAs, circRNA-mediated regulatory mechanisms in chicken muscle development have received limited research attention.

Overall, a substantial number of muscle-related lncRNAs and circRNAs remain undiscovered. We collected breast muscle samples from chickens exhibiting divergent breast muscle percentage phenotypes and performed whole-transcriptome sequencing to profile mRNA, circRNA, and lncRNA expression. Utilizing weighted gene co-expression network analysis (WGCNA) and correlation analysis, we constructed an ncRNA-mRNA regulatory network and identified key regulatory ncRNAs (e.g., MSTRG.9172.1, novel_circ_009419). These findings provide a scientific basis for poultry genetic improvement and meat yield enhancement.

## 2. Results

### 2.1. LncRNA/circRNA Profiling of Breast Muscle in Chicken

**Figure 1:**
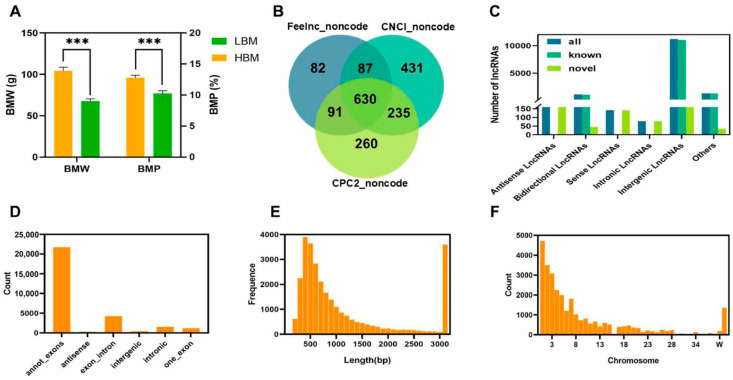
Overview of lncRNA and circRNA expression in breast muscle. (**A**) Graph of expression differences between high- and low-breast-muscle samples. *** indicated *p* < 0.001. BMW—breast muscle weight; BMP—breast muscle percentage; LBM—low breast muscle; HBM—high breast muscle. (**B**) Identification of novel lncRNAs in breast muscle. CPC2_noncode—Coding Potential Calculator 2_noncode; CNCI_noncode—Coding-Non-Coding Index_noncode; Feelnc_noncode—FlExible Extraction of LncRNAs_noncode. (**C**) Statistics for lncRNA numbers based on different types. (**D**) Statistics for circRNA numbers based on different types. (**E**) Distribution of length of circRNAs. (**F**) Distribution of circRNA in each chromosome of the chicken.

To explore the functions of ncRNAs in regulating muscle development, we measured the body weight (BM) and breast muscle weight (BMW) and calculated the breast muscle percentage (BMP) of Xianju (XJ) chickens. Considering the correlation between BM and BMW, the 12 samples were categorized into high-breast-muscle (HBM) and low-breast-muscle (LBM) groups based on BMP trait (Figure 1A and Figure S1), and whole-transcriptome sequencing of the muscle samples was conducted. More than 140 Gb of clean data points were filtered, and more than 70% of them were aligned to the genome exon regions. After alignment, coding ability prediction, and quantification, a total of 17,109 mRNAs (16,012 known mRNAs and 102 novel mRNAs), 14,087 lncRNAs (13,457 known lncRNAs and 630 novel lncRNAs), and 29,410 circRNAs were annotated and predicted.

For lncRNAs, we used three methods to predict the coding ability and screen novel non-coding transcripts. As mentioned above, a total of 630 novel lncRNAs were identified (Figure 1B), including 142 sense lncRNAs and 160 antisense lncRNAs. The lncRNAs were located predominantly in intergenic regions (Figure 1C).

Using CIRIquantv software (1.1.2) for circRNA identification, a total of 21,715 circRNAs were identified from exons, and 4246 circRNAs were identified from exon—intron regions (Figure 1D). A total of 33.48% of the circRNAs were 300–500 bp in length (Figure 1E). On different chromosomes, there were 15,549 circRNAs from chromosomes 1 to 5, accounting for 53.13% (Figure 1F).

### 2.2. Identification of Candidate lncRNAs Affecting Breast Muscle Development

**Figure 2:**
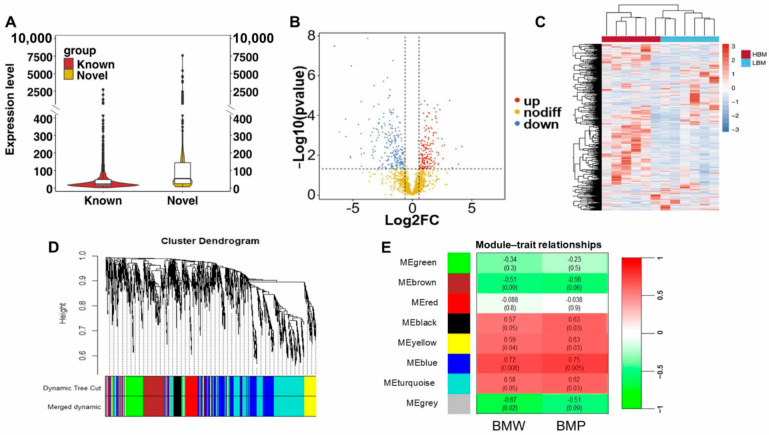
Differential expression analysis and functional prediction of lncRNAs. (**A**) Comparison of expression levels between known and novel lncRNAs. (**B**) Volcano plot of differentially expressed lncRNAs. (**C**) Expression pattern of differentially expressed lncRNAs. The group and lncRNA type were annotated; the right part indicated the fold change in expression. (**D**) Clustering and merging results for gene modules in WGCNA pipeline. (**E**) The relationship between BMW/BMP and lncRNAs modules.

To identify candidate lncRNAs affecting breast muscle development, we selected lncRNAs with an average transcripts per million (tpm) expression greater than 1 for subsequent analysis, resulting in a total of 1419 lncRNAs. The expression of known and novel lncRNAs was quantified, and the results revealed that the expression of novel lncRNAs was significantly greater than that of known lncRNAs (Figure 2A). Differentially expressed lncRNAs (DElncRNAs) were subsequently identified by DESeq2 between the HBM and LBM groups, and 390 DElncRNAs were screened (upregulated—217, downregulated—173) (Table S1, Figure 2B). Different expression patterns of DElncRNAs were obtained using hierarchical clustering methods (Figure 2C). To infer the gene network associated with breast muscle development, WGCNA was performed based on breast muscle traits and lncRNA expression (tpm > 1), and the soft threshold (β = 7) was determined when R^2^ > 0.85. A total of eight modules were obtained (Figure 2D,E), and 59~419 lncRNAs were found in each module (Table S2). According to the threshold (r > 0.6 or r < −0.6, *p* < 0.05), the blue module was determined to be significantly and positively associated with BMW and BMP, of which 95 lncRNAs were identified based on |Key Module Eigengene-based connectivity| (|KME|) > 0.8 (Table S3). In combination with DElncRNAs, 51 candidate lncRNAs were screened, including 12 novel lncRNAs (Table S3).

### 2.3. Screening for Candidate circRNAs Affecting Breast Muscle Development

**Figure 3:**
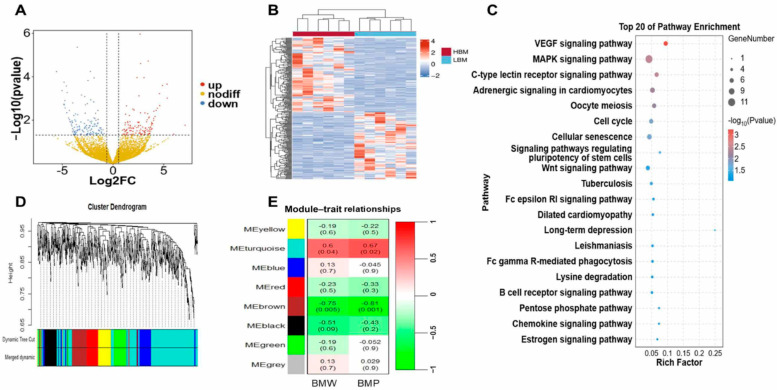
Differential expression analysis and functional prediction of lncRNAs. circRNAs differential expression analysis and functional prediction. (**A**) Volcano plot of differentially expressed circRNAs. (**B**) Expression pattern of differentially expressed circRNAs. (**C**) KEGG enrichment based on the source genes of DEcircRNAs. (**D**) Clustering and merging results for gene modules in WGCNA pipeline. (**E**) The relationship between BMW/BMP and circRNAs modules.

Similarly to the pipeline of lncRNA selection, we selected circRNAs with an average reads per million mapped (rpm) expression greater than 100 for subsequent analysis, resulting in a total of 897 circRNAs. A total of 262 circRNAs (upregulated—138, downregulated—124) were differentially expressed between the high- and low-breast-muscle phenotypes (Figure 3A, Table S4). The hierarchical cluster analysis of the differentially expressed circRNAs (DEcircRNAs) revealed that the expression profiles of the samples in the HBM and LBM groups could be categorized into distinct clusters (Figure 3B). We performed a Kyoto Encyclopedia of Genes and Genomes (KEGG) functional analysis of the source genes from the DEcircRNAs to explore their biological functions. Ten important pathways were significantly enriched, including the MAPK signaling pathway and the Wnt signaling pathway (Figure 3C, Table S5). To infer the gene network associated with breast muscle development, WGCNA was performed on the basis of breast muscle traits and circRNA expression (rpm > 100), and a total of eight modules were obtained (Figure 3D,E); 70~399 circRNAs were found in each module (Table S6). The brown and turquoise modules were determined to be significantly associated with breast weight and the breast rate based on thresholds (r > 0.6 or r < −0.6, *p* < 0.05) (Table S7). Forty-six genes with |KME| > 0.8 in both modules were selected and combined with DEcircRNAs to screen for 22 candidate circRNAs (Table S7).

### 2.4. Identification of Hub Genes and Construction of lncRNA/circRNA-mRNA Networks

**Figure 4:**
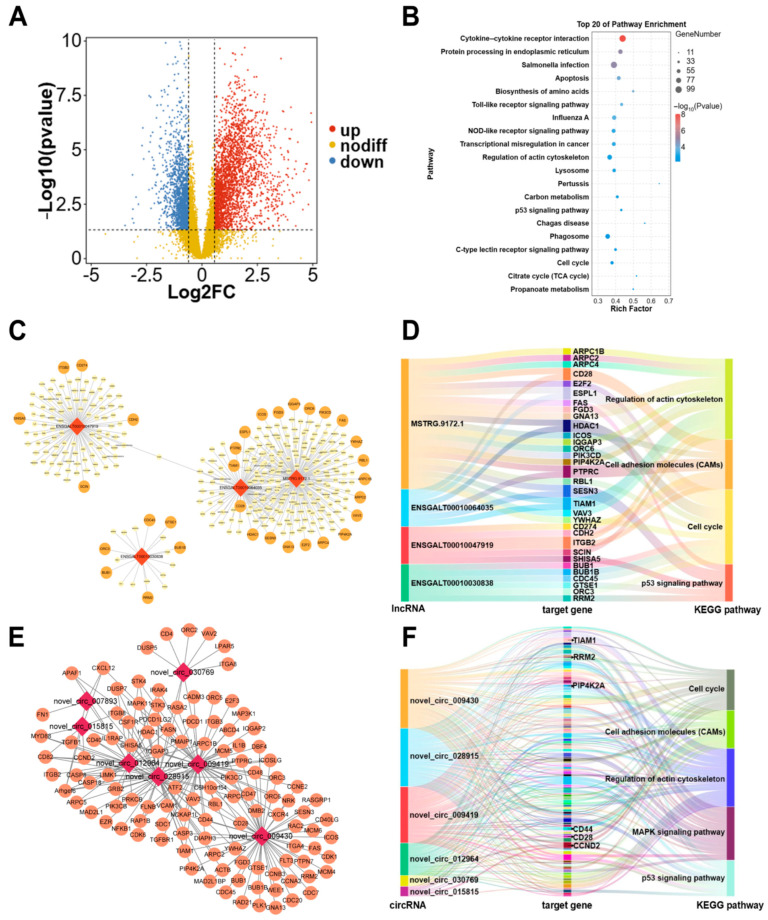
Identification of hub genes and construction of lncRNA/circRNA-mRNA networks. (**A**) Volcano plot of differentially expressed mRNAs. (**B**) KEGG enrichment based on DEGs. (**C**) LncRNA–DEGs regulatory network based on the trans, cis, and antisense methods. Diamond squares indicate candidate lncRNAs and larger circles indicate target genes of candidate lncRNAs. (**D**) Relationship of candidate lncRNAs and their target genes to the KEGG pathway. (**E**) CircRNA-DEGs regulatory network based on Pearson’s correlation analysis. Diamond squares indicate candidate circRNAs and larger circles indicate target genes of candidate circRNAs. (**F**) Relationship of candidate circRNAs and their target genes to the KEGG pathway.

The competitive endogenous RNA (ceRNA) mechanism relies on the correlations between the transcript levels of different molecule pairs (e.g., lncRNA–mRNA, circRNA–mRNA) across numerous samples. To identify candidate mRNA functions associated with breast muscle development, we selected mRNAs with an average tpm > 1 for differential expression gene (DEG) analysis, and we identified 4181 DEGs (upregulated—2564, downregulated—1617) between the two groups (Figure 4A, Table S8). The KEGG pathway analysis revealed a total of 41 significantly different pathways, including those related to the regulation of the actin cytoskeleton, p53 signaling pathway, and other pathways (Figure 4B, Table S9). Next, for the candidate lncRNAs, we analyzed the target genes via three approaches, namely, trans, cis, and antisense target analyses. DEGs and 51 candidate lncRNAs were selected for analysis, and trans regulation was defined when the correlation coefficient was >0.95 and *p* < 0.001. A total of 665 lncRNA–DEG pairs were identified in the trans relationship (Figure 4C, Table S10). Additionally, 16 and 2 lncRNA—DEG pairs were identified in cis and antisense relationships, respectively (Figure 4C, Table S10). The KEGG enrichment analysis of all target genes revealed significant enrichment in the cell adhesion molecules, cell cycle, regulation of the actin cytoskeleton, and p53 signaling pathway, which were closely related to muscle development. Based on the KEGG enrichment of genes from each lncRNA, we found that MSTRG.9172.1, ENSGALT00010030838, and ENSGALT00010047919 were related to muscle growth (Figure S2). Among which, MSTRG.9172.1 is associated with 21 target genes (e.g., *PIP4K2A*, *TIAM1*, and *CD28*) involved in these pathways. In addition, ENSGALT00010064035 may also play a crucial role in muscle development, because the candidate genes (e.g., *CD28* and *TIAM1*) were regulated by ENSGALT00010064035 (Figure 4D).

For circRNAs, 2589 circRNA—DEG pairs (correlation coefficient > 0.95, *p* < 0.001) were identified by screening DEGs and 22 candidate circRNAs using Pearson’s correlation analysis (Figure 4E and Figure S3, Table S11). Similarly to lncRNA, the KEGG enrichment analysis of the target genes revealed significant enrichment in the MAPK signaling pathway, cell adhesion molecules, cell cycle, regulation of the actin cytoskeleton, and p53 signaling pathway, which is closely related to muscle development. Seven candidate circRNA-DEG networks were selected due to the functions of target genes (e.g., *PIP4K2A*, *TIAM1*, and *CD44*), including novel_circ_009419, novel_circ_009430, novel_circ_12964, novel_circ_15815, novel_circ_028915, novel_circ_030769, and novel_circ_007893 (Figure S4). Among which, novel_circ_009419, novel_circ_009430, and novel_circ_028915 were more closely associated with muscle development, as over 50 target genes within these pathways were regulated by these circRNAs (Figure 4F).

### 2.5. Validation of Candidate lncRNAs/circRNAs and mRNAs

**Figure 5:**
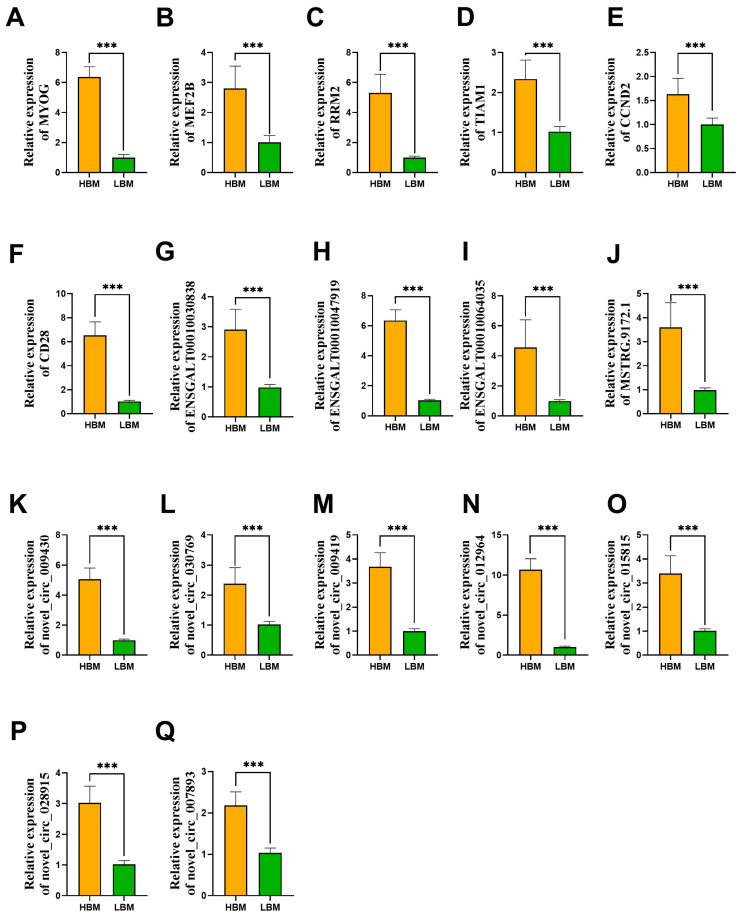
Validation of ncRNA sequencing by RT-PCR. (**A**–**F**) Expression of *MYOG*, *MEF2B, RRM2*, *TIAM1*, *CCND2*, and *CD28* between the HBM and LBM groups. (**G**–**J**) Expression of candidate lncRNAs between the HBM and LBM groups. (**K**–**Q**) Expression of candidate circRNAs between the HBM and LBM groups. *** indicated *p* < 0.001.

To confirm the expression levels of the sequenced RNA molecules, two genes, *MYOG* and *MEF2B*, were selected to validate the muscle phenotype-based grouping strategy. We found that *MYOG* and *MEF2B* were positively related to BMP and BMW in the sequenced and expanded populations, indicating the validity of the grouping method used in this study. Next, we selected candidate target genes (*RRM2*, *TIAM1*, *CCND2*, and *CD28*), lncRNAs (ENSGALT00010030838, ENSGALT00010047919, ENSGALT00010064035, and MSTRG.9172.1), and circRNAs (novel_circ_009430, novel_circ_030769, novel_circ_009419, novel_circ_012964, novel_circ_015815, novel_circ_007893, and novel_circ_028915) to confirm transcript abundance in the expanded population (Figure 5). The results revealed that the candidate genes *TIAM1*, *CCND2*, *RRM2*, and *CD28* were significantly highly expressed in the HBM group, and the results of the four candidate lncRNAs and seven candidate circRNAs were consistent with those of the transcriptome, which were positively correlated with *TIAM1*, *CCND2*, *RRM2*, and *CD28*. Additionally, the expression of genes related to the cell cycle was further examined. *PCNA* and *CCND1* were not differentially expressed between the two groups based on the mRNA sequencing results. However, the real-time PCR (RT-PCR) results of *PCNA* and *CCND1* revealed borderline significant differences between the HBM and LBM groups (*p* < 0.1). The relative expression of *P21* is significantly reduced in the HBM group, while the relative expression of *CCNE2* and *CDK2* is significantly increased in the HBM group (Figure S5).

## 3. Discussion

Chicken meat, a vital source of high-quality animal protein, is the most consumed meat globally [22]. As a major component of chicken skeletal muscle, breast muscle exhibits moderate heritability for weight traits, providing a theoretical basis for enhancing breast muscle yield through genetic approaches [23]. Tan et al. implemented genomic selection for breast muscle trait improvement, revealing heritability estimates of 0.24–0.38 for both BMW and BMP. Three generations of selection increased BMW from <400 g to >500 g and improved BMP from <20% to 22% [23]. Subsequent research identified the *SOX6* gene as a key regulator of breast muscle development, showing a significant correlation with wooden breast occurrence [24]. The discovery of key molecular markers has accelerated selection for breast muscle traits. The intramuscular fat content in the muscle tissue of female XJ chickens is higher than that of roosters, and their flavor compounds are more abundant, resulting in a superior meat quality and taste compared with males, making them more popular among consumers. XJ chickens reach a market weight of 1.4–1.7 kg at the market age (D140), with a feed conversion ratio > 3. During sample collection, we selected the thickest portion of the right pectoralis major muscle as the sampling area. This anatomical region represents the most representative site for chicken breast muscle research, and this selection criterion is consistent with established methodologies reported in published transcriptomic, metabolomic, and proteomic studies of chicken breast muscle [25,26,27]. Our study focuses on epigenetic molecular markers that influence breast muscle development, providing lncRNA, circRNA, and mRNA expression profiles from muscle tissues with divergent phenotypes. Regulatory network analysis identified key ncRNAs. Although their expression trends with target genes were verified, their efficacy as molecular breeding markers requires further validation.

Both lncRNAs and circRNAs constitute essential components of the regulatory circuitry governing skeletal muscle growth and development [9,28,29]. Mechanistically, lncRNAs can function as ceRNAs, sequestering miRNAs to alleviate the miRNA-mediated repression of target mRNAs [30]. This interaction results in positive correlations between lncRNA and mRNA expression patterns. In this study, muscle development-related genes (e.g., *BCL6*, *MYOG*, and *MEF2B*) exhibited significantly elevated expressions in the HBM group. Jawasreh et al. demonstrated a significant positive correlation between body weight and *MYOG* expression levels in muscular tissues across chickens with divergent body weights [31], which is consistent with our findings. Analytical methods such as WGCNA and genome-wide association study can link genes to phenotypes, significantly narrowing down the range of candidate genes, thereby improving the efficiency of screening for key functional genes and accelerating the development of molecular breeding markers [32,33]. Our study focused on lncRNA WGCNA. This approach identified key modules associated with breast muscle traits and facilitated the construction of regulatory networks to pinpoint crucial lncRNAs. Furthermore, we identified four pivotal lncRNAs that primarily regulate target genes (e.g., *TIAM1*, *SESN3*, and *RRM2*), modulating muscle development through pathways including the p53 signaling pathway and the regulation of the actin cytoskeleton. Notably, *TIAM1* activity can be mediated by syndecan-4 to influence myoblast migration [34]. *SESN3*, a stress-responsive gene, is significantly upregulated during human primary myotube differentiation, and its silencing increases myostatin expression [35]. RRM2, a crucial subunit of the ribonucleotide reductase (RNR), affects RNR activity and DNA replication. *RRM2* overexpression activates the Hippo–YAP signaling pathway, promoting cardiomyocyte proliferation [36,37]. In poultry, *RRM2* overexpression significantly enhances myoblast viability and proliferation while inhibiting differentiation and skeletal muscle regeneration [38]. Consistent with these findings, *TIAM1*, *SESN3*, and *RRM2* expression was significantly elevated in the HBM groups. Moreover, MSTRG.9172.1 regulates the highest number of target genes (197/683), suggesting its potential importance as a novel lncRNA in muscle development regulation.

CircRNAs are widely distributed in the nucleus and cytoplasm, where they regulate source gene expression and function as miRNA sponges to counteract the miRNA-mediated suppression of target genes [18,39]. The analysis of DEcircRNA source genes revealed a significant enrichment of the MAPK signaling pathway, which plays a critical regulatory role in skeletal muscle growth and development. For instance, the p38 MAPK pathway promotes satellite cell myogenic differentiation, the JNK MAPK pathway inhibits muscle differentiation, and the MAPK/ERK pathway regulates muscle fiber type transition [40,41,42]. Core MAPK components (e.g., *NFATC3*, *ATF2*, and *MAPK3*) are essential for muscle development. Specifically, *NFATC3* knockdown reduces muscle fiber number and mass [43]. MAPK3, a key component of growth factor signaling at the cell membrane, promotes myofiber type conversion and improves fatigue resistance [44]. In this study, the average expression of circRNAs derived from *NFATC3* and *MAPK3* was significantly elevated in the HBM group, suggesting that the effects of these genes on muscle development may be mediated by circRNAs. Key circRNAs (e.g., novel_circ_009430) were identified to have an important role in muscle development by regulating target genes (e.g., *CD82*, *CCND2*, *CXCR4*, and *TIAM1*), which were crucial components of the regulation of the actin cytoskeleton, p53 signaling pathway, and other related pathways. *CD82*, associated with muscular dystrophy, inhibits myoblast proliferation when suppressed [45]. The observation of higher *CD82* expression in the HBM group corroborates the above conclusion. CCND2, a cyclin promoting cell cycle progression, exhibits downregulation that significantly enhances the myogenic differentiation of progenitor cells and promotes muscle regeneration in dystrophin-deficient mice upon transplantation [46,47]. In addition to *CCND2*, the expression patterns of genes such as *P21*, *CCND1*, and others indicate that the cell cycle process in the HBM group is accelerated and the cell proliferation process is activated. *CXCR4* regulates myoblast migration via SDF-1α binding, with deficiency causing myocyte fusion defects [48]. Similarly to *CD82*, *CCND2* and *CXCR4* showed an elevated HBM expression and a significant association with novel_circ_009430, novel_circ_009410, and others, indicating their collective importance in breast muscle development. Notably, novel_circ_009419—regulating the most target genes (611/2589)—emerged as a pivotal circRNA for muscle development.

Through the investigation of ncRNAs and their target genes, our study demonstrates that the p53 signaling pathway and the regulation of the actin cytoskeleton are the most critical pathways influencing muscle development. The p53 functions as an inhibitor of myogenin to prevent nuclear abnormalities and maintain the integrity of terminally differentiated muscle cells [49]. Previous studies have reported that *SESN1* can regulate the p53 signaling pathway through a feedback mechanism, thereby promoting the proliferation of chicken myoblasts [50]. Ju et al. conducted circRNA sequencing across distinct muscle fiber types in chickens, revealing the significant enrichment of DEcircRNAs in the p53 signaling pathway [51]. These findings suggest that the p53 signaling pathway may participate in muscle fiber growth through circRNA-mediated regulatory mechanisms. The actin cytoskeleton regulates cellular motility by mediating protrusion, adhesion, contraction, and retraction [52]. A recent study reported that circRNAs regulate thigh muscle development via the regulation of the actin cytoskeleton pathway [2], which is highly consistent with our results. Collectively, non-coding RNAs modulate muscle development by regulating key components in the p53 signaling pathway, the regulation of the actin cytoskeleton, and other pathways, though more detailed mechanisms warrant further investigation.

## 4. Materials and Methods

### 4.1. Animals and Sample Collection

One hundred 1-day-old female XJ chickens were randomly selected for rearing at Sishan Farm (Hangzhou, Zhejiang Province, China). The XJ chickens were fed corn—soybean diets containing 11.43 MJ/kg metabolizable energy (ME) and 16% crude protein (CP). Fresh feed and water were available ad libitum. After 12 h of fasting, the XJ chickens were slaughtered on day 140. Traits such as BW and BMW were measured post slaughter. The individuals were divided according to their breast muscle percentage into an HBM phenotype group and an LBM phenotype group (*n* = 20), and the remaining chickens were categorized into an intermediate breast muscle group. Six samples were randomly selected for the HBM and LBM phenotype groups. The 12 samples from the thickest portion of the right pectoralis major muscle used for sequencing were snap frozen in liquid nitrogen and stored at −80 °C.

### 4.2. Total RNA Isolation and Detection

Total RNA was extracted using TRIzol kits (Invitrogen, Carlsbad, CA, USA). Concentration and integrity were determined using a Nanodrop^®^ Series Spectrophotometer ((Thermo Fisher Scientific Inc., Waltham, MA, USA)) and Agilent^®^ 2100 Bioanalyzer (Agilent Technologies Inc., Santa Clara, CA, USA), respectively, and examined using RNase-free agarose gel electrophoresis. After the extraction of total RNA, a Ribo-Zero™ Gold kit was used to remove rRNA from the total RNA, retaining only mRNA and ncRNA.

### 4.3. Library Preparation and Sequencing of Non-Coding RNAs

The enriched mRNAs and ncRNAs were dissected into fragments and reverse transcribed with random primers. The cDNA was purified and end-paired using a QiaQuick PCR kit with poly(A) and universal junctions. The cDNA was then digested, and the product size was selected using agarose gel electrophoresis, amplified using PCR, and sequenced using the Illumina NovaSeq 6000 platform.

### 4.4. Quality Control, rRNA Mapping and Removal, and Genome Alignment

The raw sequencing data were first filtered using fastp software (0.18.0) (-q 20 -u 50 -N 15 -l 50) [53,54] to remove reads containing splice sequences, excessive proportions of N bases, all A bases, and more than 50% of low-quality bases. The filtered reads were then compared to the ribosomal RNA database via Bowtie2 [55] to remove rRNA. HISAT2 v2.2.1 software (-rna-strandness RF) [56] was then used to compare the qualified reads to the chicken reference genome (GRCg7b). For mRNAs, transcripts were reconstructed using StringTie v2.1.6 software [57], and genes that were identified in this sequencing result but not included in the reference genome were defined as novel genes. Moreover, gene quantification was performed by calculating the tpm value of the mRNAs on the basis of the raw read count.

### 4.5. Prediction and Quantification of Novel lncRNAs

The transcripts were assembled using StringTie v2.1.6 [57] software. Transcripts meeting the criteria of >200 bp in length and ≥2 exons were retained. Those subsequently annotated in the GRCg7b genome were designated as known lncRNAs. For transcripts not annotated in GRCg7b genome, their coding potential was predicted using three tools: CPC2 (default parameters) [58], CNCI (-s 0 -l 200 -e 2) [59], and FEELnc (-s 200) [60]. Transcripts consistently predicted as non-coding by all three tools were classified as novel lncRNAs. lncRNAs were categorized into five groups based on their location relative to protein-coding genes: intergenic, bidirectional, intronic, antisense, and positive-sense lncRNAs. The tpm values of the lncRNAs were calculated based on the original read counts for subsequent analysis.

### 4.6. Identification and Quantification of circRNAs

After the reads were compared to the reference genome, circRNA identification was performed using CIRIquant (library type: 2: read1 matches the antisense strand; at least one sample had back-splicing junction_reads ≥ 2; length ≤ 100 kb) [61]. Novel circRNAs were defined as those unidentifiable in the circBase or circBank database, whereas the known circRNAs corresponded to known entries within these databases. CircRNAs were typed according to position and exon type, such as annotated_exons, antisense, exon_intron, intergenic, intronic, and one_exon. The distribution of circRNAs on chromosomes and the length distribution were summarized. RPM values were used to scale reverse splicing junction reads and quantify circRNA abundance.

### 4.7. Detection of Differentially Expressed Genes/Non-Coding RNAs

The gene expression levels of protein-coding genes and ncRNAs were normalized using DESeq2 [62], and differentially expressed genes (DEGs, DElncRNAs, and DEcircRNAs) were identified based on raw transcript counts (|FC| > 1.5, *p* < 0.05).

### 4.8. Weighted Gene Co-Expression Network Analysis

To identify genes associated with breast muscle development, a co-expression network derived from lncRNA and mRNA transcript abundance in breast muscle was constructed using WGCNA [63]. The circRNA soft threshold (β = 4) was determined based on the scale-free distribution results (R^2^ > 0.85). The lncRNA soft threshold (β = 7) was determined according to the results of the scale-free distribution (R^2^ > 0.85). Next, stepwise and dynamic cutting methods were applied to construct the co-expression network and detect gene modules using the following parameters: minModuleSize = 50 and mergeCutHeight = 0.25. A correlation coefficient > 0.5 was set as the threshold for the breast muscle-associated gene module, and having a |KME| > 0.8 was defined as a hub gene.

### 4.9. Functional Enrichment Analysis

To explore the potential biological functions and pathways of candidate DEGs and ncRNA target genes, we selected candidate ncRNAs with more than 15 target genes and performed KEGG analysis and visualization using the online tool OmicShare (https://www.omicshare.com/).

### 4.10. Construction of the CeRNA Network for lncRNA/circRNA–mRNA Pairs

Network construction was based on potential functional relationships between lncRNA-mRNAs and circRNA-mRNAs. For lncRNAs, Pearson’s correlation analysis was conducted to detect the target genes in trans. Genes located on the same strand within 100 kb of lncRNAs were considered cis target genes, while those on the opposite strand were regarded as antisense target genes. For CircRNA, target genes were detected using Pearson’s correlation analysis. The networks were visualized using Cytoscape 3.10 [64].

### 4.11. Validation of Candidate lncRNAs, circRNAs, and mRNAs

To verify the sequencing results, we performed real-time PCR (RT—PCR) to determine the relative expression of selected genes and ncRNAs (*MYOG*, *MEF2B*, *RRM2*, *CCND2*, *TIAM1*, *CD28*, *PCNA*, *CCND1*, *P21*, *CCNE2*, *CDK2*, ENSGALT00010030838, ENSGALT00010047919, ENSGALT00010064035, MSTRG.9172.1, novel_circ_009430, novel_circ_030769, novel_circ_009419, novel_circ_012964, novel_circ_015815, novel_circ_007893, and novel_circ_028915). The specific primers used were designed using Oligo 6.0 software, and the primer information is shown in Supplementary Table S12. The data were analyzed using the 2^−ΔΔCT^ method, with *RPL32* and *HSPA2* used as internal reference genes. RT-PCR was performed using ten independent biological replicates to ensure statistical robustness

### 4.12. Statistical Analysis

The *t*-test was used to examine the differences in the expression levels of phenotypes, ncRNAs, and mRNAs, while Pearson’s correlation analysis was employed to explore the target genes of ncRNAs. All statistics were performed via SPSS 25.0 software. A *p* value less than 0.05 indicated a statistically significant difference.

## 5. Conclusions

We generated muscular expression profiles of lncRNAs, circRNAs, and mRNAs from HBM and LBM samples and constructed an ncRNA–mRNA network to explore the mechanisms affecting breast muscle development. We identified four lncRNAs (ENSGALT00010030838, ENSGALT00010047919, ENSGALT00010064035, and MSTRG.9172.1) and seven circRNAs (novel_circ_009430, novel_circ_030769, nov-el_circ_009419, novel_circ_012964, novel_circ_015815, novel_circ_007893, and nov-el_circ_028915) as major regulatory molecules that play important roles in modulating the expression and function of various genes. These genes include *RRM2*, *CCND2*, *TIAM1*, and *CD28*, which are involved in the regulation of the actin cytoskeleton and non-coding signaling pathway.

## Data Availability

The lncRNA sequenced reads generated in this study have been submitted to the NCBI SRA database with BioProject: PRJNA1281837.

## Supplementary Materials

The following supporting information can be downloaded at: https://www.mdpi.com/article/10.3390/ijms26178181/s1.
