# Supplementary figures and images for: Novel Insights into the Molecular Mechanisms of Chicken Breast Muscle Development by Integrating Non-Coding RNA and mRNA Profiles
Source: Int J Mol Sci. 2025 Aug 23;26(17):8181. doi: 10.3390/ijms26178181 (PMC12428033; doi:10.3390/ijms26178181)

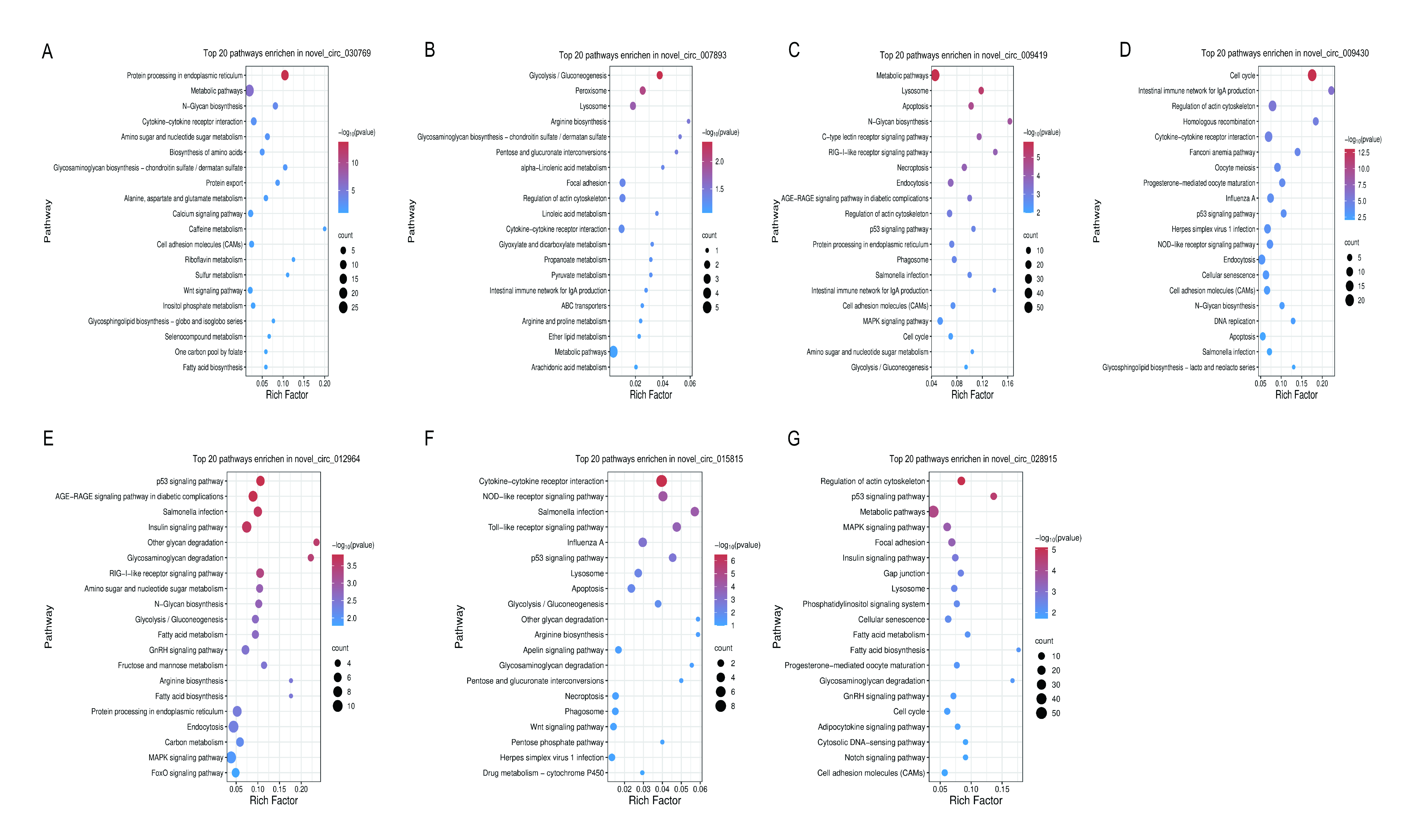

Supplement: Supplementary file 1 [file ijms-26-08181-s001.zip › Figure S1.jpg]

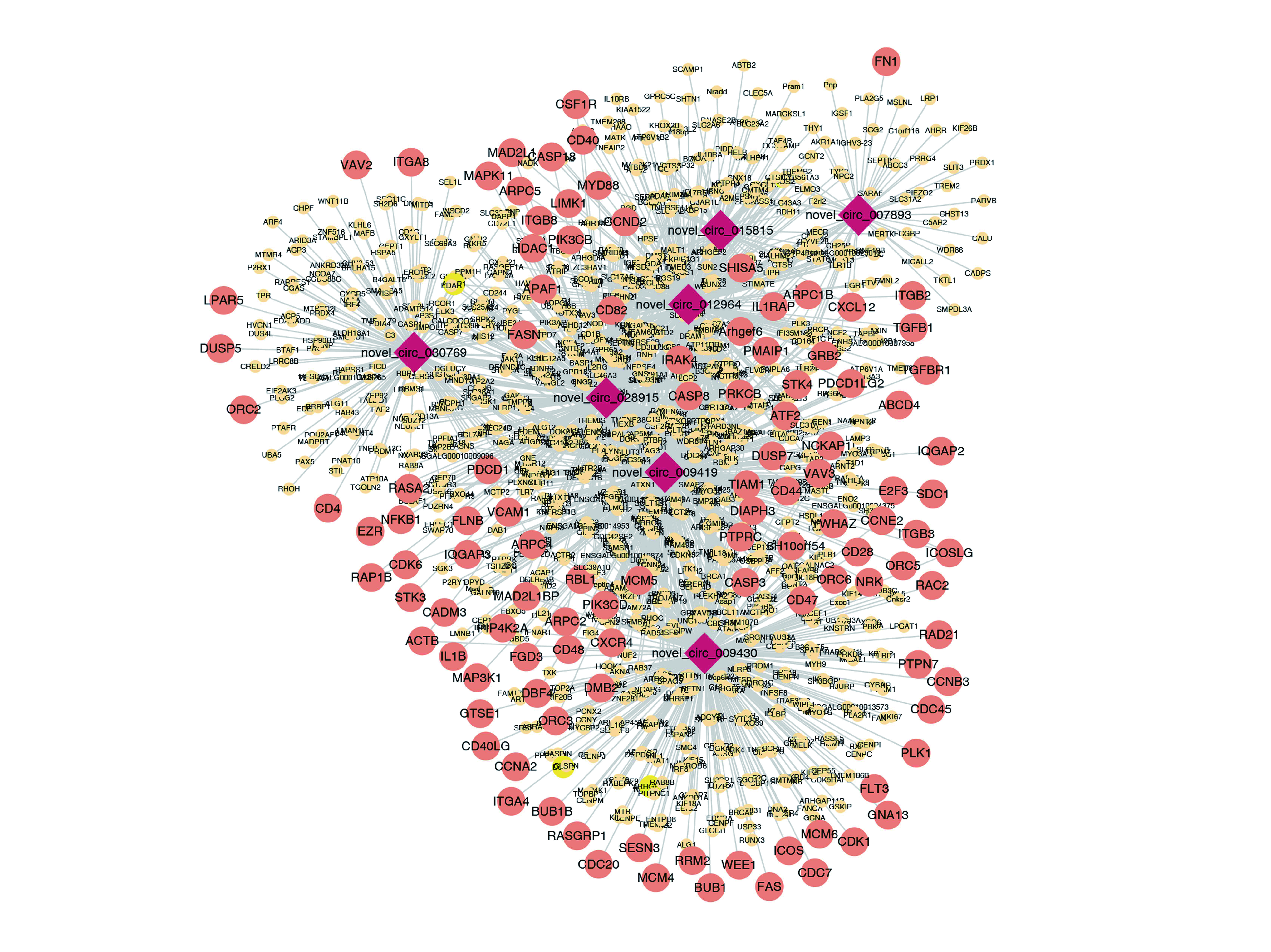

Supplement: Supplementary file 1 [file ijms-26-08181-s001.zip › Figure S2.jpg]

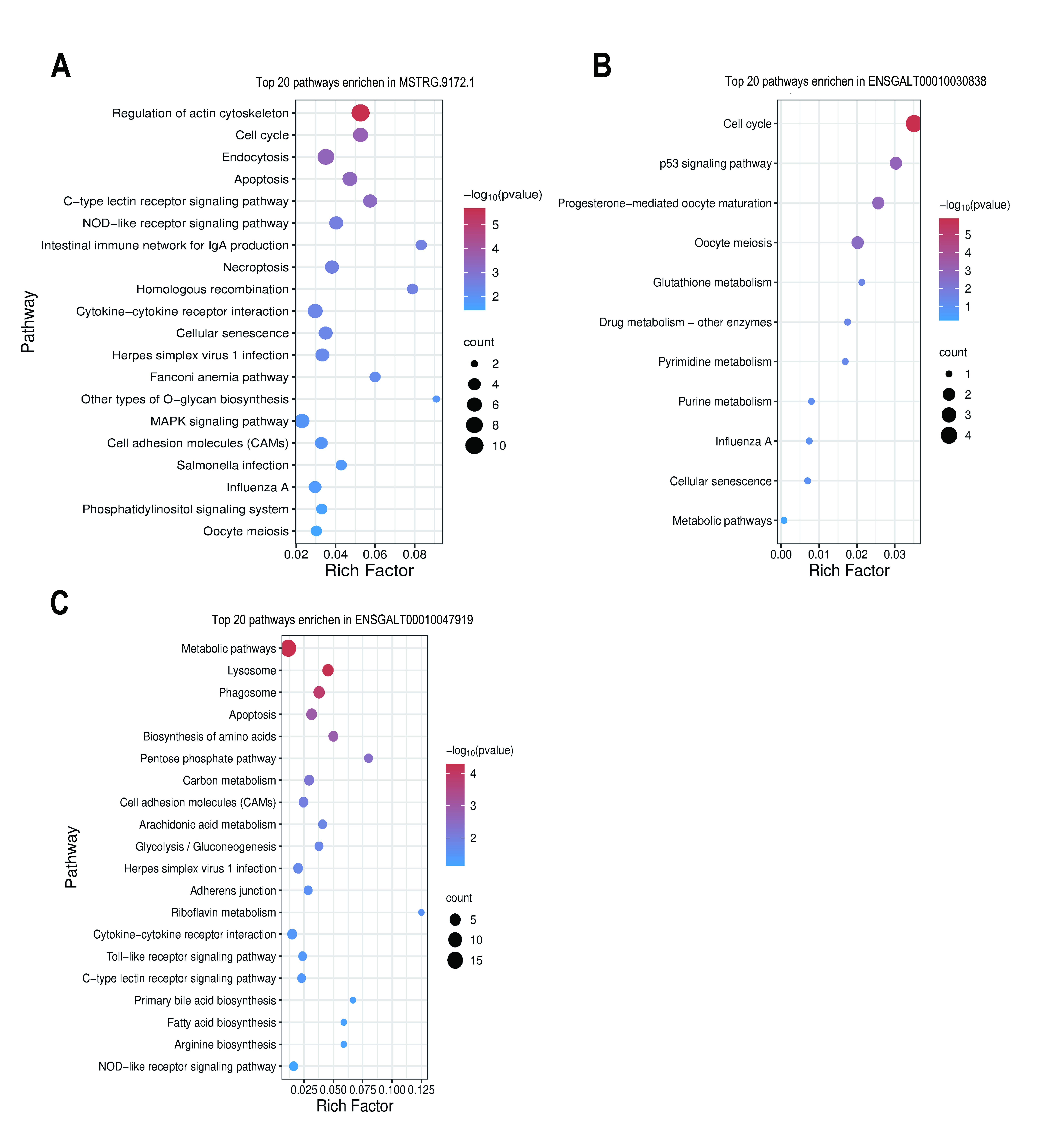

Supplement: Supplementary file 1 [file ijms-26-08181-s001.zip › Figure S3.jpg]
